# Urban Natural Environments, Obesity, and Health-Related Quality of Life among Hispanic Children Living in Inner-City Neighborhoods

**DOI:** 10.3390/ijerph13010121

**Published:** 2016-01-12

**Authors:** Jun-Hyun Kim, Chanam Lee, Wonmin Sohn

**Affiliations:** Department of Landscape Architecture and Urban Planning; Texas A&M University, College Station, TX 77843, USA; clee@arch.tamu.edu (C.L.); wonmin.sohn@tamu.edu (W.S.)

**Keywords:** health-related quality of life, urban natural environments, children, landscape spatial patterns, obesity, green space, GIS, remote sensing, landscape ecology

## Abstract

Although a substantial body of literature has provided evidence supporting the positive effects of natural environments on well-being, little has been known about the specific spatial patterns of urban nature in promoting health-related quality of life (HRQOL) among children. This study assessed the association that the urban natural environment measured by landscape spatial patterns may have with obesity and HRQOL among Hispanic children. Ninety-two 4th and 5th grade students were recruited from Houston, Texas, and the Pediatric Quality of Life Inventory (PedsQL) was used to capture the children’s HRQOL. The quality of urban natural environments was assessed by quantifying the landscape spatial patterns, using landscape indices generated by Geographic Information Systems and remote sensing. From the bivariate analyses, children’s body mass index showed a significantly negative association with their HRQOL. After controlling for socio-demographic factors, the results revealed that larger and more tree areas were positively correlated with children’s HRQOL. In addition, those children living in areas with tree patches further apart from each other showed higher HRQOL. This research adds to the current multi-disciplinary area of research on environment-health relationships by investigating the roles of urban greeneries and linking their spatial structures with children’s HRQOL.

## 1. Introduction

Studies connecting neighborhood environments with public health outcomes have grown rapidly in recent years. Earlier works have focused on how natural and well-designed built environments can support physical activities such as walking and bicycling [1,2,3,4,5,6,7,8,9,10,11,12,13,14]. In addition, many previous studies have examined the relationship between urban natural environments and mental health condition [15,16]. Researchers found that urban greeneries can contribute to public health, not only by promoting outdoor activities, but also by helping people relieve daily stresses through their well-documented restorative effects [15,17,18]. However, although a substantial body of literature has provided evidence supporting the effects of the natural environment on the well-being of adult populations, the influence of the natural environment on the physical and mental health outcomes of children or adolescents has not yet been fully investigated. In addition, so far, little is known about the role of urban natural environments shaping landscape spatial patterns in promoting health-related quality of life (HRQOL), especially among children and adolescents. The effects of the natural and built environments on children’s health require more investigation because the results of childhood environmental interactions will likely differ from those of adult populations [19].

A number of previous studies have focused on the relationship between the natural environment and children’s mental health. Wells and Evans [20] examined the effects of nearby natural environments in rural residential areas on children’s psychological well-being. With the 337 rural 3rd to 5th grade children studied, they found that living near nature could offer moderating effects on the impacts of stressful life events, and also improve cognitive functioning. In addition, children living in greener residential environments showed lower levels of stress from life events, while children living in environments with little nearby nature reported higher levels of stress. Kuo and Faber Taylor [21] examined the influence of natural environments on Attention Deficit Hyperactivity Disorder (ADHD) in children. They found that ADHD symptoms were significantly reduced during green outdoor activities than in activities conducted in either built outdoor or indoor settings. In addition, this result appeared consistent across a wide range of characteristics including individual demographic factors, residential settings, and ADHD case severity. Amoly and colleagues [22] also observed 2111 children from 7 to 10 years of age and examined the associations among green spaces, children’s behavioral development, and symptoms of ADHD. By using the Normalized Difference Vegetation Index (NDVI) to examine green spaces, they found that both the accessibility and usage times of green spaces surrounding children’s residential areas were associated with decreased behavioral problems, including ADHD. Similar cross-sectional studies showed how urban green spaces could play a pivotal role in soothing children’s hyperactivity and behavioral problems [23,24]. A small number of studies emphasized the effects of the built environment on children’s HRQOL. After reviewing extensive literature on pediatrics, Sherman and colleagues [25] synthesized certain components of the physical environment that influence children’s mental health, including their psychological, emotional, and cognitive functioning. They found access to nature to be beneficial in improving children’s HRQOL. Although many pediatric researchers have recognized the potential impacts of nature on children’s HRQOL, it has not yet fully been measured and a significant knowledge gap still exists.

Childhood obesity caused by a sedentary lifestyle is strongly associated with a poor quality of life and low level of well-being [26,27,28,29]. The majority of studies on childhood and adolescent quality of life have focused on groups with chronic health conditions or comorbidities. Several studies have attempted to measure the HRQOL of obese children or adolescents [26,27,28,29,30,31,32,33]. Schwimmer and colleagues [32] recruited three groups of children and adolescents: an obese group, a healthy group, and a group of children and adolescents diagnosed with cancer. Of these groups, obese children and adolescents showed significantly lower values of HRQOL than did healthy children and adolescents. Obesity in a child or adolescent increased the likelihood of an impaired HRQOL 5.5 times more than a healthy child or adolescent, and 1.3 times more than a cancer patient. In addition, the authors stated that measuring HRQOL of Hispanic children would be important due to their high risk of obesity. Tyler and colleagues [34] supported the conclusion that obese Mexican-American children showed lower HRQOL than other ethnicity groups. Studies involving obese adults have shown that they tend to have a lower HRQOL than non-obese adults [35,36], but more empirical studies are necessary that examine HRQOL and obese children [32]. Since children are one of the most vulnerable population groups as they may not be ready to make informed health-related choices by themselves, more studies are required to understand the influence of obesity on their quality of life. Because evidence suggests that childhood obesity could be related to many psychological and social factors [37], the multidimensional and comprehensive structure of HRQOL makes it a useful tool for assessing and enhancing childhood health conditions [32,38]. 

Based on these findings, the main purpose of this research is to examine the association between urban natural environments measured by landscape spatial patterns and HRQOL among Hispanic children. Since it is widely known that natural environments in neighborhoods provide positive effects on improving the mental health of adults [18,39,40,41] and children [20,21], it was hypothesized that children’s HRQOL would be positively associated with the quality of their neighborhood environments and landscape spatial patterns. In addition, this research hypothesized that children’s HRQOL would be negatively correlated with their levels of obesity.

## 2. Methods 

### 2.1. Study Location and Sample

To obtain reliable self-reported data [42], this research recruited ninety-two 4th and 5th grade students (9 to 11 years old) from five elementary schools in the East End district of Houston, Texas. The East End district is located on the east side of the Houston downtown area. The rationale in choosing this district was as follows. First, according to 2010 Census data [43], a significant proportion of this area is Hispanic (85%) with children younger than 18 years of age (27%). Second, 32% of East End households reported earning less than $ 20,000 in 2010, and 30% of Hispanics in the district reported living in poverty. In addition, 48% of residents who are 25 years and older had no high school diploma, and only 12% had a college degree or higher. Finally, the district offers diverse physical environmental settings including different types of parks, land uses, and housing types. As the neighborhoods in this district were established in the early 1910s, many mature trees have shaped the landscape spatial patterns.

### 2.2. Data Collection and Measurement

The consent forms, protocols, and data collection for this study were approved by the Institutional Review Board (IRB) at both the University of Houston and Texas A&M University (2008-0092). Our IRB approval process reviewed every item of this study including all instructions of the Declaration of Helsinki. A research staff administered surveys to participating children at each school site, capturing all of the study variables for this research. This study used the specific study eligibility criteria to recruit participants: children being of Hispanic origin who can speak and read in either English or Spanish, and children who do not have any medical restrictions limiting their physical activity. All subjects voluntarily signed their informed consent for participating in this research.

#### 2.2.1. Health-Related Quality of Life (Dependent Variable)

To evaluate children’s HRQOL, this research used the Pediatric Quality of Life Inventory (PedsQL^TM^) 4.0 generic core scale developed by Varni [38,44,45,46,47,48]. According to Varni [38], the PedsQL is a modular instrument used to assess HRQOL among children and adolescents ages 2 to 18. The PedsQL consists of parallel child self-reports and parent proxy reports in essentially identical forms. Separate reports from parents and children are used because children’s self-reports are designed to assess perceptions of their internal states, while parents’ reports reflect their children’s observable behaviors. This instrument has been tested extensively and used in many HRQOL studies [29,32,33,34]. Most questions regarding perceptions related to health conditions use a five-point Likert scale (0 = never a problem, 1 = almost never a problem, 2 = sometimes a problem, 3 = often a problem, and 4 = almost always a problem). The 23-item PedsQL 4.0 generic core scale is classified into four sub-factors, including physical functioning (8 items), emotional functioning (5 items), social functioning (5 items), and school functioning (5 items). To facilitate the description of children’s HRQOL (higher scores show better HRQOL), after collecting all of the questionnaires, each item was reverse-scored and linearly transformed on a zero to 100 scale (0 = 100, 1 = 75, 2 = 50, 3 = 25, 4 = 0). Then, a total scale score calculated by the mean of all 23 items was determined to indicate a summary of the child’s HRQOL. Across the ages, the total scale score of self-reporting and proxy-reporting was shown to approach a high score Cronbach alpha reliability coefficient of 0.90 [38]. Previous research has shown that the PedsQL can detect HRQOL differences between healthy and unhealthy children [32,38,44]. The PedsQL was selected for this research because of its high reliability and ease of use for both participants and researchers. 

#### 2.2.2. Landscape Spatial Patterns (Independent Variables)

To quantify the quality of the urban natural environments in children’s neighborhoods, this research utilized the patch-corridor-matrix model (P-M model), which has been widely used in the landscape ecology field to measure landscape spatial patterns [49]. To classify land cover, 1m color infrared (CIR) high-resolution Digital Orthophoto Quarter Quadrangle (DOQQ) images from the National Agriculture Imagery Program (NAIP) were obtained from the Texas Natural Resources Information System (TNRIS) [50]. To objectively measure different land cover types, Geographic Information Systems (GIS; ArcGIS Version 9.2; ESRI, Redlands, CA, USA) and remote sensing (ENVI Version 4.3, geospatial image analyzing software; ITT Visual Information Solutions, White Plains, New York, NY, USA) were used. Based on light spectral similarities with the unsupervised classification process, 40 different land cover classes were generated. Those classes were then grouped into three main land cover types: grass areas, trees/forests, and developed/impervious areas. To remove isolated pixels affecting the landscape index values, post-classification processes were conducted; these included sieving, clumping, and filtering [51,52]. 

**Figure 1:**
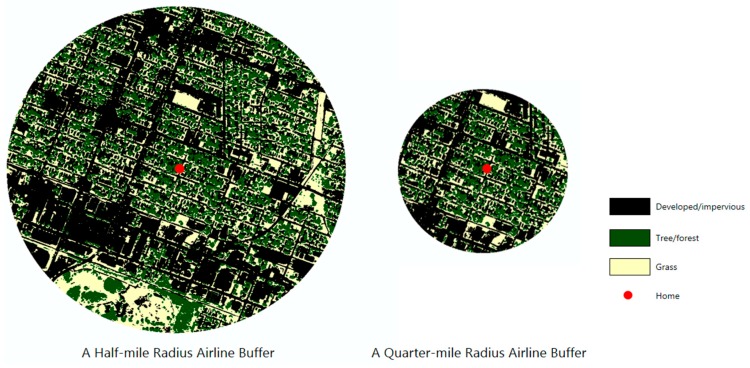
Different spatial settings to measure landscape spatial patterns.

For measuring landscape spatial patterns surrounding a participant’s home, both half-mile and quarter-mile airline buffers (Figure 1) were generated and analyzed using various landscape indices obtained from FRAGSTATS 3.3, a spatial pattern analysis software program developed by McGarigal and Marks [53]. The buffer distance of a half-mile, approximately 800 m, around homes was selected based on the reported distance that both adults and children would likely be willing to walk from previous research [54,55,56,57]. Based on previous studies, a quarter-mile buffer, approximately 400 m, was also added to assess the more proximate home neighborhood environments around each child’s home [58,59]. In addition, this research tested the associations between the children’s HRQOL and landscape spatial patterns of urban forests and trees within the half-mile and quarter-mile street network buffers. However, the PedsQL scores were not associated with any landscape indices captured within these buffer types. 

From the greater body of landscape indices that measure landscape spatial patterns, this research selected six landscape indices: Percentage of Landscape (PLAND), Number of Patches (NP), Mean Patch Size (MPS), Mean Shape Index (MSI), Mean Nearest Neighbor Distance (MNN), and Patch Cohesion Index (COHESION) (Table 1). To select the appropriate landscape indices, we reviewed the previous literature and established guidelines/criteria addressing both landscape ecological and public health perspectives [12,39,49,60,61,62,63,64,65]. Each landscape index quantified various aspects of the urban natural environment, including configurations such as size and the existence of tree patches, shapes of patch boundaries, distances between single patches, and connectivity. 

#### 2.2.3. Control Variables

Control variables including socio-demographic, children’s Body Mass Index (BMI), physical activity, and environmental perception variables were captured from the surveys. To collect the children’s BMI data, we objectively collected students’ heights and weights using a scale (TANITA TBT 310) and portable stationmaster (Seca 213). Each participant’s height and weight was measured twice, and the average of the two assessments was used for the final value. Participants’ heights and weights were then used to calculate their BMI values. The Quetelet’s index (weight in kg/height m^2^) was used to compute the BMI. The guidelines provided by the Center for Disease Control and Prevention (CDC) [66] were applied to analyze age- and gender-specific BMI z-scores. The Physical Activity Questionnaire for Older Children (PAQ-C) [67] was used to capture the children’s physical activity levels in different situations including both weekday and weekend days, and sedentary activity patterns. Participants’ neighborhood environmental perceptions were captured regarding accessibility, safety, comfort, attractiveness, and satisfaction, using a survey based on previously-developed instruments [68,69,70,71] and the relevant literature [1,57,72].

**Table 1:** Selected landscape indices, acronyms, formulas, and descriptive statistics of landscape spatial patterns.

Criteria	Landscape Indices (Acronym)	Formula ^a^	Description	Unit (Range)	Mean *	Std. Dev.
*Size & Existence & Fragmentation*	Percentage of landscape (PLAND)	$\sum_{j=1}^{a}a_{ij}/A\times 100$	Higher PLAND values indicate larger patch sizes.	%	25.99	7.29
	Number of patches (NP)	$n_{i}$	Higher NP values indicate more number of patches and fragmented condition.	Count	3809.29	1211.09
	Mean Patch size (MPS)	$\sum_{j=1}^{n}a_{ij}/n_{i}$	Lower MPS values indicate more fragmented conditions.	Square-meter (MPS ≥ 0, without limit)	162.54	128.06
*Shape*	Mean shape index (MSI)	$\left[\sum_{j=1}^{n}\left(0.25p_{ij}/\sqrt{a_{ij}}\right)\right]/n_{i}$	Higher MSI values indicate more irregular shapes.	None (MSI ≥ 1, without limit)	1.25	0.03
*Distance (Isolation)*	Mean nearest neighbor distance (MNN)	$\sum_{j=1}^{a}h_{ij}/n_{i}$	Higher MNN values indicate more isolated patterns.	Meter	2.87	0.39
*Connectivity*	Patch cohesion index (COHESION)	$\begin{array}{l} \left(1-\sum_{j=1}^{n}p_{ij}/\sum_{j=1}^{n}\left(p_{ij}\sqrt{a_{ij}}\right)\right)\times \\ {\left(1-1/\sqrt{A}\right)}^{-1}\times 100 \end{array}$	Higher COHESION values indicate more connected patterns.	%	97.78	1.15

Notes: This table is adopted and revised form Kim *et al.* [12]. *n*_i_ = number of patches in the landscape of patch type I; *a*_ij_ = area (m^2^) of patch ij; A = total landscape area (m^2^); *p*_ij_ = perimeter of patch ij; *h*_ij_ = distance (m) from patch ij to nearest neighboring patch of the same type, based on edge-to-edge distance. * Mean values of half-mile airline buffers; **^a^** See McGarigal and Marks [53] for more details.

### 2.3. Data Analysis

The data analysis focused on assessing the correlations between landscape spatial patterns of urban nature and children’s HRQOL. First, descriptive statistics were performed to understand the respondents’ socio-demographic characteristics, overall physical activity patterns, BMI, HRQOL, and landscape spatial patterns computed by landscape indices. Then the standard diagnostic testing was performed to identify key variables and outliers. In addition, this study tested for the potential multicollinearity problems among the independent variables, especially with regards to the various landscape indices. Once descriptive analyses were completed, bivariate analyses were conducted to detect associations between independent variables (landscape spatial patterns) and the dependent variable (children’s HRQOL). Correlations among children’s BMI and HRQOL were also evaluated. 

A Principal Component Analysis (PCA) was performed to reduce the number of environmental perception variables. The criterion for selecting the appropriate number of factors was above the 0.5 value of the Kaiser-Myer-Olkin (KMO) and Bartlett’s test of sphericity with the varimax rotation [73,74]. This research also suppressed absolute values of factor loading less than 0.4 [75]. Finally, statistical models were estimated using a series of multiple regression analyses to predict children’s HRQOL, using the independent variables of the landscape indices and controlling for BMI, physical activity, factor scores of environmental perception, and socio-demographic variables. Separate models were estimated using the GIS variables captured at different buffer sizes. We used SPSS 19.0 (IBM Corp., Armonk, NY, USA) to analyze the data. 

## 3. Results

### 3.1. Characteristics of the Participants

Table 2 shows the socio-demographic characteristics of the participants. The proportion of the children’s genders was approximately 40% boys and 60% girls. Their ages ranged between 9 and 11 years old. In terms of ethnicity, the Hispanic group was dominant with over 80%; the rest of the respondents answered “don’t know” to queries regarding their ethnicities. The majority of the participants was born in the U.S. and living with both parents. The average age of the respondents’ mothers was about 36 years old. Approximately 65% of the mothers were married. The majority of the mothers were employed, followed by those identifying as homemakers; only about 20% had at least a college degree. The children lived in households with an average of five people. The majority had at least two TVs and two cars per household. About 58% of the children were categorized as overweight or obese, and their mean BMI z-score was 0.90. 

**Table 2:** Socio-demographic characteristics of child respondents (*N* = 92).

Variables	Freq. (%)	Variables	Freq. (%)	Variables	Freq. (%)
**Gender**		**Mother’s age**	**Value**	**People in Household**	**Value**
Boy	35 (38.0%)	Mean	35.98	Mean	5.11
Girl	57 (62.0%)	SD	7.17	SD	1.60
**Age**		**Mother’s marital status**		**# of TVs at Home**	
9	21 (22.8%)	Single, never married	13 (14.1%)	0	1 (1.1%)
10	50 (54.4%)	Married	60 (65.2%)	1	9 (9.8%)
11	21 (22.8%)	Living with partner	7 (7.6%)	2	22 (23.9%)
**Grade**		Divorced, widow, separated	11 (12.0%)	3 +	60 (65.2%)
4th	43 (46.7%)	Other	1 (1.1%)		
5th	49 (53.3%)	**Mother’s employment**		**Number of cars per household**	
**Ethnicity**		Employed for wages	39 (42.4%)	0	5 (5.4%)
Hispanic	76 (82.6%)	Self-employed	6 (6.5%)	1	33 (35.9%)
Don’t know	16 (17.4%)	Out of work for more than 1 year	5 (5.4%)	2	42 (45.7%)
**Country Born**		Our of work for less than 1 year	6 (6.5%)	3 +	12 (13.0%)
US	75 (81.5%)	A homemaker	33 (35.9%)	**Weight Status ^a^**	
Mexico	12 (13.0%)	A student	1 (1.1%)	Obese or overweight	53 (57.6%)
Central America	3 (3.3%)	Retired	1 (1.1%)	Normal or underweight	39 (42.4%)
Missing	2 (2.2%)	Unable to work	1 (1.1%)	**BMI**	**Value**
**Guardians**		**Mother’s education**		Mean	21.69
Mom only	15 (16.3%)	Elementary to 6th	15 (16.3%)	SD	5.53
Dad only	0 (0.0%)	7th–8th	12 (13.1%)	**BMI z-score**	**Value**
Mom and Dad	70 (76.1%)	9th–12th	45 (48.9%)	Mean	0.90
Parent and Step-parent	6 (6.5%)	College degree or higher	15 (16.3%)	SD	1.21
Missing	1 (1.1%)	Vocational/technical	5 (5.4%)		

**^a^** obese or overweight = at or above the 85th percentile of body mass index (BMI) values; Normal or underweight = less than the 85th percentile of BMI values.

According to their physical activity patterns, children showed a relatively low frequency of walking to utilitarian destinations including schools, parks, friends’ houses, and stores or shops. About 62% did not walk to school during a typical week. In addition, over 40% of children did not walk to a park on a weekly basis. More than 65% participated in physical education or athletics classes only once or twice a week, and only about 30% of children were engaged in physical activities twice or three times on a regular weekend day. As a result of their sedentary patterns, children reported longer amounts of time watching TV during a typical weekend, as compared to weekdays. About 38% of children watched TV more than three hours per weekday, while approximately 52% spent more than three hours watching TV on the weekends. Overall, respondents shared a generally positive environmental perception of and satisfaction with their neighborhoods. In using a five-point Likert scale, approximately 51% of children believed they had adequate accessibility to recreational or playable places, and more than 58% were satisfied with walking or biking conditions in and around their neighborhoods.

### 3.2. Children’s HRQOL 

Table 3 represents the children’s HRQOL measured by both the children’s self-report and their mothers’ proxy PedsQL surveys. The children’s self-reported PedsQL scores were higher than the scores assessed by their mothers. This result supports the findings of previous research [31]. The total PedsQL score was composed of two main health summary scores: the physical and psychosocial health summary scores; both summary scores were higher in the children’s self-report surveys as compared to the mothers’ proxy surveys. In the children’s self-report surveys, boys reported higher total PedsQL scores (1799.29) than girls (1726.79), while girls had higher total PedsQL scores (1664.91) than boys (1624.24) in the mothers’ proxy reports. However, a child’s gender did not play a statistically significant role in either survey.

**Table 3:** Children’s health-related quality of life (HRQOL) Assessed by the pediatric quality of life (PedsQL) Surveys.

Surveys	Statistics	Mean PedsQL Score	Total PedsQL Score	Physical Health Summary Score	Psychosocial Health Summary Score
Child self-report survey	Mean	76.35	1754.67	632.61	1122.25
	Std. Deviation	13.32	307.86	131.96	214.72
Mother proxy survey	Mean	71.44	1650.00	554.62	1093.33
	Std. Deviation	16.41	377.75	187.52	237.37

Total of mean PedsQL score = 100, total of the total PedsQL score = 2300, total of the physical health summary score = 800, total of the psychosocial health summary score = 1500.

The results of the bivariate analyses of the children’s BMI and HRQOL indicated that the children’s BMI z-scores were negatively correlated to their HRQOL. According to the unadjusted bivariate analysis results from the children’s self-report PedsQL surveys, there was a negatively significant relationship between the children’s BMI z-scores and their physical health summary scores (*p* < 0.05), while the total PedsQL scores were not significantly associated with their BMI. On the other hand, there were slightly more statistically significant correlations between the children’s BMI z-scores and their total and psychosocial health summary scores from the mothers’ proxy surveys (Table 4).

**Table 4:** Correlations between children’s BMI and HRQOL.

Variables	Child Self-Report Survey		Mother Proxy Survey	
	Beta	Sig.	Beta	Sig.
Total PedsQL score	−0.146	0.166	−0.248	0.019 *
Physical health summary score	−0.227	0.030 *	−0.165	0.117
Psychosocial health summary score	−0.068	0.520	−0.269	0.010 *

Notes: Dependent variable: Children’s BMI z-score. * *p* < 0.05.

### 3.3. Correlation between Children’s HRQOL and Landscape Spatial Patterns

This research estimated two multivariate regression models to measure the association between landscape spatial patterns and children’s HRQOL (Table 5). The first model (HA) was based upon the half-mile radius airline buffer measures, and the other model (QA) was based upon the quarter-mile radius airline buffer measures. The HA model explained about 43% of the variance in the children’s HRQOL measured by the self-reported PedsQL scores. The children’s HRQOL was negatively associated with their age (*p* < 0.05). Their HRQOL was positively correlated with the mother’s employment status, but the p value was .061. The children’s HRQOL was negatively correlated with their BMI z-scores (*p* = 0.01) and weekend television watching times (*p* < 0.05), while more weekend physical activity time was more likely to lead to a higher HRQOL value (*p* < 0.01). Among the variables related to neighborhood environmental perceptions, neighborhood disorder (*p* < 0.01) and walking barriers (*p* < 0.05) were negatively related to children’s HRQOL, whereas accessibility to schools and open spaces to play (*p* < 0.05) and the existence of parks in their neighborhoods (*p* < 0.01) were positively associated with their HRQOL. Regarding the landscape spatial pattern variables, PLAND (*p* < 0.05), NP (*p* < 0.05), and MNN (*p* < 0.01) were positively correlated with the children’s HRQOL. Other landscape indices (MPS and MSI) showed a negative relationship with the respondent’s HRQOL, but only of marginal significance (0.05 < *p* < 0.1). These results indicated that children having larger (higher PLAND) and more (higher NP) tree/forest areas within a half-mile of their homes were likely to have higher HRQOL. In addition, longer distances between tree patches (higher MNN) were positively associated with higher HRQOL. 

The QA model using the quarter-mile radius airline buffer measured the children’s HRQOL to be approximately 42%. Among the socio-demographic variables, only age was negatively associated with the children’s PedsQL scores. Similar to the results of the HA model, the children’s BMI z-scores (*p* < 0.01) and weekend television watching times (*p* < 0.01) were negatively related to their HRQOL, whereas their physical activity times during a regular weekend day (*p* < 0.01) were positively correlated with their HRQOL. From the neighborhood environment perception variables, neighborhood disorder (*p* < 0.01) was negatively associated with the children’s HRQOL. However, accessibility to schools and open spaces to play (*p* < 0.05) and the existence of parks in the neighborhood (*p* < 0.05) were both positively correlated with improved HRQOL. Among the selected landscape indices, NP (*p* < 0.05) and MNN (*p* < 0.01) showed significantly positive relationships with the children’s HRQOL, which was consistent with the HA model results. The QA model results suggest that children living in areas with more tree patches and longer distances between the patches were likely to report higher HRQOL. 

**Table 5:** Landscape Indices Correlated with children’s HRQOL: Final Regression Model Results Based on the Half-Mile Airline (HA) and Quarter-mile Airline (QA) Buffer Measures.

Half-Mile Airline Buffer: Model HA			Quarter-Mile Airline Buffer: Model QA		
Variables	Beta	Sig.	Variables	Beta	Sig.
**Socio-Demographic Factors**			**Socio-Demographic Factors**		
Children’s age	−0.189	0.041 *	Children’s age	−0.243	0.010 *
Children’s gender ^a^	0.202	0.038 *	Children’s gender ^a^	0.182	0.061
Mother’s employment status ^b^	0.170	0.061	Mother’s employment status ^b^	0.139	0.142
**BMI and Physical Activity (PA)**			**BMI and Physical Activity (PA)**		
Children’s BMI z-score	−0.239	0.010 *	Children’s BMI z-score	−0.247	0.008 **
Total weekend PA times	0.392	0.000 **	Total weekend PA times	0.346	0.000 **
Total TV watching hours during the weekend	−0.225	0.015 *	Total TV watching hours during the weekend	−0.252	0.005 **
**Neighborhood Environmental Perceptions**			**Neighborhood Environmental Perceptions**		
Accessibility for utilitarian walking	0.163	0.065	Accessibility to playgrounds and streets to play	0.171	0.059
Unattractiveness in walking conditions	−0.142	0.099	Neighborhood disorder	−0.283	0.002 **
Neighborhood disorder	−0.279	0.002 **	Accessibility to schools and open spaces to play	0.194	0.041 *
Walking barriers	−0.201	0.035 *	Park existence in neighborhoods	0.251	0.015*
Accessibility to schools and open spaces to play	0.198	0.042 *			
Park existence in neighborhoods	0.298	0.005 **			
**Landscape Spatial Patterns**			**Landscape Spatial Patterns**		
PLAND	0.357	0.023 *	PLAND	0.255	0.069
NP	0.382	0.016 *	NP	0.385	0.020 *
MPS	−0.299	0.072	MPS	N/S	N/S
MSI	−0.191	0.089	MSI	N/S	N/S
MNN	0.608	0.001 **	MNN	0.536	0.004 **
COHESION	N/S	N/S	COHESION	N/S	N/S
(Constant: Coeff. = 3060.916 **)			(Constant: Coeff. = 2848.380 **)		
N = 92/Sig. < 0.000 / Adj. R^2^ = 0.431			N = 92/Sig. < 0.000 / Adj. R^2^ = 0.423		

Dependent Variable: Child Self-report Total PedsQL Score. Dummy variables: **^a^** 0 = girl, 1 = boy, **^b^** 0 = unemployment, 1 = employment. Abbreviations: PLAND, percentage of landscape; NP, number of patches; MPS, mean patch size; MSI, mean shape index; MNN, mean nearest neighbor distance; COHESION, patch cohesion index; N/S, not significant. More detailed information about each landscape index in Table 1. * *p* < 0.05; ** *p* < 0.01.

## 4. Discussion

This research assessed the correlations between landscape spatial patterns and HRQOL among Hispanic children. Although a number of researchers reported that there were significant correlations between the natural environment and mental health conditions [15,17,18,39,40,41,65,76], only a few studies have attempted to assess relationships related to objectively-measured landscape spatial patterns of urban natural environments. This research expands the existing knowledge regarding those relationships. The results revealed that larger sizes (PLAND) and a greater number (NP) of forests or tree areas in urban neighborhoods were positively associated with Hispanic children’s HRQOL. In addition, longer distances between tree/forest patches (MNN) were positively associated with children’s HRQOL. These results may relate to safety concerns about urban natural environments. A sense of safety in open spaces has been found to be significantly related to HRQOL [77]. In previous studies, a sense of safety was associated with landscape structure [63,78,79], and they found more open spaces and clearer edge conditions without dense understories would be considered to be safer than closed configurations [63,80]. Thus, the MNN’s positive association with HRQOL can be explained by the fact that higher MNN values typically represent landscape spatial patterns with sufficient openings between patches. In addition, this research found that more regularly shaped landscape patterns (lower MSI) were positively associated with children’s HRQOL. Although it was a marginal significance between MSI and children’s HRQOL, this conclusion also supports the notion that clearly delineated edges contribute to children’s higher HRQOL. 

The final models for this study showed consistent results with those of previous studies indicating negative relationships among BMI, physical activity, and HRQOL [35−37]. The level of physical activity and overall physical health strongly affect mental health conditions and human well-being. Many researchers have documented that being more active could provide a better sense of mental well-being [37,81]. In addition, previous research has reported that obese children and adults present significantly lower values of HRQOL than those of normal weight [32,35,36]. However, few studies have considered the influence of urban natural environments on children’s HRQOL and BMI. The results of this research have confirmed that there are significant correlations between children’s BMI, level of physical activity, and HRQOL as they relate to neighborhood landscape spatial patterns [29,31,82,83,84,85,86]. 

This study has several limitations. First, because this study captured a large number of variables through labor-intensive recruitment and data collection methods to ensure completeness and accurate matches of child-mother pairs, only a relatively small number of samples (*n* = 92 pairs) could be recruited; therefore, not all potential confounding factors could be considered in the multivariate modeling. Second, the study focused on an inner-city district with a high proportion of minorities and economically challenged populations; therefore, the results are not generalizable to other settings or populations. Third, this research did not objectively measure children’s physical activity; conversely, children’s BMI and landscape spatial patterns were evaluated using objective measurements. Finally, to examine the landscape spatial patterns of urban forests and trees, this research used DOQQ imagery. This imagery only allows for the analysis of two-dimensional landscape patterns determined by different land cover types. To understand the full range of layers in landscape structure, more advanced media such as LIDAR (Light Detection and Ranging) satellite imagery should be considered. Future studies should consider a larger sample size, and additional and more diverse population groups and community settings. Despite these limitations, to our knowledge, this is one of the first empirical studies assessing associations between children’s HRQOL, BMI, and landscape spatial patterns shaped by urban trees and forests, captured objectively through landscape indices. 

## 5. Conclusions

This research adds to the current multi-disciplinary research on environment-health relationships by investigating the roles of urban greeneries and linking their spatial structures to Hispanic children’s HRQOL. Hispanic populations possess more serious conditions related to physical inactivity and obesity, and they are more likely to be economically challenged, and suffer from poverty and limited access to healthcare. Many previous studies documented that Hispanic children and adolescents were more vulnerable to health conditions such as a high rate of sedentary patterns, obese conditions, and an impaired quality of life than other ethnicity groups [32,34]. The better quality of urban neighborhood environments with more green spaces would be a feasible means of promoting physical activity and improving quality of life by incorporating the practice into children’s daily routines. One of the critical goals in urban and landscape planning is improving the quality of life of community members. Because landscape spatial patterns provide one of the most important factors shaping neighborhood environments and have been shown to improve psychological and physical health, it should be considered as a significant neighborhood component requiring proper planning and management. To improve the quality of life among children, it is essential to understand the specific roles of various natural and built environment elements within their neighborhoods. Further, to promote quality of life among children living in urban communities, continued multi-disciplinary efforts are needed to incorporate urban nature and ecological planning considerations into the decision-making processes in the urban planning and public health fields.

## Abbreviations

HRQOL: Health-related quality of life
ADHD: Attention Deficit Hyperactivity Disorder
PedsQL^TM^: Pediatric Quality of Life Inventory
DOQQ: Digital Orthophoto Quarter Quadrangle
GIS: Geographic Information Systems
PLAND: Percentage of Landscape
NP: Number of Patches
MPS: Mean Patch Size
MSI: Mean Shape Index
MNN: Mean Nearest Neighbor Distance
COHESION: Patch Cohesion Index
BMI: Body Mass Index
HA: Half-mile airline
QA: Quarter-mile airline
NDVI: Normalized Difference Vegetation Index

## References

[1] Jago R., Baranowski T., Baranowski J.C. (2006). Observed, GIS, and self-reported environmental features and adolescent physical activity. Am. J. Health Promotion.

[2] Brownson R.C., Hoehner C.M., Day K., Forsyth A., Sallis J.F. (2009). Measuring the built environment for physical activity: State of the science. Amer. J. Prev. Med..

[3] Giles-Corti B., Broomhall M.H., Knuiman M., Collins C., Douglas K., Ng K., Lange A., Donovan R.J. (2005). Increasing walking: How important is distance to, attractiveness, and size of public open space?. Am. J. Prev. Med..

[4] Giles-Corti B., Donovan R.J. (2003). Relative influences of individual, social environmental, and physical environmental correlates of walking. Am. J. Public Health.

[5] King A.C., Stokols D., Talen E., Brassington G.S., Killingsworth R. (2002). Theoretical approaches to the promotion of physical activity: Forging a transdisciplinary paradigm. Am. J. Prev. Med..

[6] Lee C., Moudon A.V. (2004). Physical activity and environment research in the health field: Implications for urban and transportation planning practice and research. J. Plan. Lit..

[7] Moudon A., Lee C., Cheadle A.D., Garvin C., Johnson D.B., Schmid T.L., Weathers R.D. (2007). Attributes of environments supporting walking. Amer. J. Health Promotion.

[8] Moudon A.V., Lee C., Cheadle A.D., Garvin C., Johnson D., Schmid T.L., Weathers R.D., Lin L. (2006). Operational definitions of walkable neighborhood: Theoretical and empirical insights. J. Phys. Act. Health.

[9] Saelens B.E., Handy L.S. (2008). Built environment correlates of walking: A review. Med.Sci. Sport. Exerc..

[10] Saelens B.E., Sallis J.F., Black J.B., Chen D. (2003). Neighborhood-based differences in physical activity: An environment scale evaluation. Am. J. Public Health.

[11] Tilt J.H., Unfried T.M., Roca B. (2007). Using objective and subjective measures of neighborhood greenness and accessible destinations for understanding walking trip and bmi in Seattle, Washington. Am. J. Health Promot..

[12] Kim J.-H., Lee C., Olvara N.E., Ellis C.D. (2014). The role of landscape spatial patterns on obesity in hispanic children residing in inner-city neighborhoods. J. Phys. Act. Health.

[13] Booth K.M., Pinkston M.M., Poston W.S.C. (2005). Obesity and the built envrionment. J. Am. Diet. Assn..

[14] Norman G.J., Nutter S.K., Ryan S., Sallis F.S., Calfas K.J., Patrick K. (2006). Community design and access to recreational facilities as correlates of adolescent physical activity and body-mass index. J. Phys. Act. Health.

[15] Kaplan R. (1984). Impact of urban nature: A theoretical analysis. Urban Ecol..

[16] Kaplan S., Kaplan R. (2003). Health, supportive environments, and the reasonable person model. Am. J. Public Health.

[17] Hartig T., Mang M., Evans G.W. (1991). Restorative effects of natural environment experiences. Environ. Behav..

[18] Ulrich R.S., Simons R.F., Losito B.D., Fiorito E., Miles M.A., Zelson M. (1991). Stress recovery during exposure to natural and urban environments. J. Environ. Psychol..

[19] Jackson R.J., Tester J. (2008). Environment shapes health, including children's mental health. J. Am. Acad. Child Adolesc. Psy..

[20] Wells N.M., Evans G.W. (2003). Nearby nature: A buffer of life stress among rural children. Environ. Behav..

[21] Kuo F.E., Faber Taylor A. (2004). A potential natural treatment for attention-deficit/hyperactivity disorder: Evidence from a national study. Am. J Public Health.

[22] Amoly E., Dadvand P., Forns J., López-Vicente M., Basagaña X., Julvez J., Alvarez-Pedrerol M., Nieuwenhuijsen M.J., Sunyer J. (2014). Green and blue spaces and behavioral development in barcelona schoolchildren: The breathe project. Environ. Health Perspect..

[23] Balseviciene B., Sinkariova L., Grazuleviciene R., Andrusaityte S., Uzdanaviciute I., Dedele A., Nieuwenhuijsen M.J. (2014). Impact of residential greenness on preschool children’s emotional and behavioral problems. Int. J. Environ. Res. Public Health.

[24] Markevych I., Tiesler C.M.T., Fuertes E., Romanos M., Dadvand P., Nieuwenhuijsen M.J., Berdel D., Koletzko S., Heinrich J. (2014). Access to urban green spaces and behavioural problems in children: Results from the giniplus and lisaplus studies. Environ. Int..

[25] Sherman S.A., Shepley M.M., Varni J.W. (2005). Children's environments and health-related quality of life: Evidence informing pediatric healthcare environmental design. Child. Youth Environ..

[26] De Beer M., Hofsteenge G.H., Koot H.M., Hirasing R.A., Delemarre-van de Waal H.A., Gemke R. (2007). Health-related-quality-of-life in obese adolescents is decreased and inversely related to BMI. Acta Paediat..

[27] Ottova V., Erhart M., Rajmil L., Dettenborn-Betz L., Ravens-Sieberer U. (2012). Overweight and its impact on the health-related quality of life in children and adolescents: Results from the european kidscreen survey. Qual. Life Res..

[28] Perry T.T., Moore P.C., Redwine K.M., Robbins J.M., Weber J.L. (2012). Physical activity, screen time and pediatric health-related quality of life in Mississippi delta. Open J. Prev. Med..

[29] Tsiros M.D., Olds T., Buckley J.D., Grimshaw P., Brennan L., Walkley J., Hills A.P., Howe P.R.C., Coates A.M. (2009). Health-related quality of life in obese children and adolescents. Int. J. Obesity.

[30] Pinhas-Hamiel O., Singer S., Pilpel N., Fradkin A., Modan D., Reichman B. (2005). Health-related quality of life among children and adolescents: Associations with obesity. Int. J. Obesity.

[31] Zeller M.H., Modi A.C. (2006). Predictors of health-related quality of life in obese youth. Obesity.

[32] Schwimmer J.B., Burwinkle T.M., Varni J.W. (2003). Health-related quality of life of severely obese children and adolescents. J. Am. Med. Assn..

[33] Williams J., Wake M., Hesketh K., Maher E., Waters E. (2005). Health-related quality of life of overweight and obese children. J. Am. Med. Assn..

[34] Tyler C., Johnston C.A., Fullerton G., Foreyt J.P. (2007). Reduced quality of life in very overweight mexican american adolescents. J. Adolesc. Health.

[35] Fontaine K.R., Bartlett S.J. (1998). Estimating health-related quality of life in obese individuals. Dis. Manag. Health Outcomes.

[36] Kolotkin R.L., Head S., Hamilton M., Tse C.-K.J. (1995). Assessing impact of weight on quality of life. Obesity Res..

[37] Banis H.T., Varni J.W., Wallander J.L., Korsch B.M., Jay S.M., Adler R., Garciatemple E., Negrete V. (1988). Psychological and social adjustment of obese children and their families. Child Care Health Develop..

[38] Varni J.W., Seid M., Kurtin P.S. (2001). PedsQL^TM^ 4.0: Reliability and validity of the Pediatric Quality of Life Inventory^TM^ Version 4.0 Generic Core Scales in healthy and patient populations. Med. Care.

[39] Hartig T., Johansson G., Kylin C. (2003). Residence in the social ecology of stress and restoration. J. Soc. Issues.

[40] Kaplan S. (1995). The restorative benefits of nature: Toward an integrative framework. J. Environ. Psychol..

[41] Sugiyama T., Leslie E., Giles-Corti B., Owen N. (2008). Associations of neighbourhood greenness with physical and mental health: Do walking, social coherence and local social interaction explain the relationships?. J. Epidemiol. Community Health.

[42] Welk G.J., Corbin C.B., Dale D. (2000). Measurement issues in the assessment of physical activity in children. Res. Quart. Exerc. Sport.

[43] U.S. Census Bureau American Community Survey 5-Year Estimates. http://www.census.gov/programs-surveys/acs/.

[44] Varni J.W., Seid M., Rode C.A. (1999). The PedsQL^TM^: Measurement model for the pediatric quality of life inventory. Med. Care.

[45] Chan K.S., Mangione-Smith R., Burwinkle T.M., Rosen M., Varni J.W. (2005). The PedsQL^TM^: Reliability and validity of the short-form generic core scales and asthma module. Med. Care.

[46] Varni J.W., Burwinkle T.M., Seid M., Skarr D. (2003). The PedsQL™ 4.0 as a pediatric population health measure: Feasibility, reliability, and validity. Ambul. Pediatr..

[47] Varni J.W., Seid M., Knight T.S., Uzark K., Szer I.S. (2002). The PedsQL^TM^ 4.0 generic core scales: Sensitivity, responsiveness, and impact on clinical decision-making. J. Behav. Med..

[48] Swallen K.C., Reither E.N., Haas S.A., Meier A.M. (2005). Overweight, obesity, and health-related quality of life among adolescents: The national longitudinal study of adolescent health. Pediatrics.

[49] Forman R.T.T. (1995). Land Mosaics: The Ecology of Landscapes and Regions.

[50] TNRIS Texas Natural Resources Information System. https://tnris.org/data-download/#!/county/Harris.

[51] Gong P., Mahler S.A., Biging G.S., Newburn D.A. (2003). Vineyard identification in an oak woodland landscape with airborne digital camera imagery. Int. J. Remote Sens..

[52] Mas J.-F., Gao Y., Pacheco J.A.N. (2010). Sensitivity of landscape pattern metrics to classification approaches. For. Ecol. Manag..

[53] McGarigal K., Marks B.J. (1995). Spatial Pattern Analysis Program for Quantifying Landscape Structure.

[54] Ewing R. (1995). Beyond density, mode choice, and single purpose trips. Transp. Quart..

[55] Lee C., Moudon A.V. (2006). The 3Ds + R: Quantifying land use and urban form correlates of walking. Transp. Res. Pt. D: Trans. Environ..

[56] Lee C., Moudon A.V., Courbois J.-Y.P. (2006). Built environment and behavior: Spatial sampling using parcel data. Ann. Epidemiol..

[57] Timperio A., Crawfore D., Telford A., Salmon J. (2004). Perceptions about the local neighborhood and walking and cycling among children. Prev. Med..

[58] McMillan T.E. (2007). The relative influence of urban form on a child’s travel mode to school. Transp. Res. Part A: Policy Pract..

[59] Kaczynski A.T., Henderson K.A. (2007). Environmental correlates of physical activity: A review of evidence about parks and recreation. Leisure Sci..

[60] Dramstad W.E., Olson J.D., Forman R.T.T. (1996). Landscape Ecology Principles In Landscape Architecture And Land-Use Planning.

[61] Forman R.T.T. (1995). Some general principles of landscape and regional ecology. Landscape Ecol..

[62] Shafer C., Cook E.A., van Lier H.N. (1994). Beyond park boundaries. Landscape Planning and Ecological Networks.

[63] Jorgensen A., Hitchmough J., Calvert T. (2002). Woodland spaces and edges: Their impact on perception of safety and preference. Landscape Urban Plan..

[64] Mitchell R., Popham F. (2007). Greenspace, urbanity and health: Relationships in england. J. Epidemiol. Community Health.

[65] Ulrich R.S. (1984). View through a window may influence recovery from surgery. Science.

[66] CDC Percentile Data Files with Lms Values. http://www.cdc.gov/growthcharts/percentile_data_files.htm.

[67] Crocker P.R., Bailey D.A., Faulkner R.A., Kowalski K.C., McGrath R. (1997). Measuring general levels of physical activity: Preliminary evidence for the physical activity questionnaire for older children. Med. Sci. Sport. Exerc..

[68] Brownson R.C., Chang J.J., Eyler A.A., Ainsworth B.E., Kirtland K.A., Saelens B.E., Sallis J.F. (2004). Measuring the environmental friendliness toward physical activity: A comparison of the reliability of 3 questionnaires. Amer. J. Public Health.

[69] Forsyth A., Schumitz K.H., Oakes M. Twin Cities Walking Survey. http://www.Activelivingresearch.Org/node/10619.

[70] SIP 4–99 Research Group Environmental Supports for Physical Activity Questionnaire. http://prevention.sph.sc.edu/tools/docs/Env_Supports_for_PA.pdf.

[71] Telford A., Salmon J., Jolley D., Crawford D. (2004). Reliability and validity of physical activity questionnaires for children: The children’s leisure activities study survey (class). Pediatr. Exerc. Sci..

[72] Hume C., Ball K., Salmon J. (2006). Development and reliability of a self-report questionnaire to examine children's perceptions of the physical activity environment at home and in the neighborhood. Int. J. Behav. Nutr. Phys. Activ..

[73] Hutcheson G., Sofroniou N. (1999). The Multivariate Social Scientist: Introductory Statistics Using Generalized Linear Models.

[74] Kaiser H.F. (1974). An index of factorial simplicity. Psychometrika.

[75] Stevens J.P. (1992). Applied Multivariate Statistics for the Social Sciences.

[76] Kaplan R., Kaplan S. (1989). The Experience of Nature: A Psychological Perspective.

[77] Parra D.C., Gomez L.F., Sarmiento O.L., Buchner D., Brownson R., Schimd T., Gomez V., Lobelo F. (2010). Perceived and objective neighborhood environment attributes and health related quality of life among the elderly in Bogota, Colombia. Soc. Sci. Med..

[78] Schroeder H., Anderson L. (1985). Perception of personal safety in urban recreation sites. J. Leisure Res..

[79] Sugiyama T., Ward Thompson C. (2008). Associations between characteristics of neighbourhood open space and older people's walking. Urban For. Urban Green..

[80] Ulrich R.S. (1986). Human responses to vegetation and landscapes. Landscape Urban Plan..

[81] Fox K.R. (1999). The influence of physical activity on mental well-being. Public Health Nutr..

[82] Fullerton G., Tyler C., Johnston C.A., Vincent J.P., Harris G.E., Foreyt J.P. (2007). Quality of life in mexican-american children following a weight management program. Obesity.

[83] Kolotkin R.L., Zeller M., Modi A.C., Samsa G.P., Polanichka Quinlan N., Yanovski J.A., Bell S.K., Maahs D.M., Gonzales de Serna D., Roehrig H.R. (2006). Assessing weight-related quality of life in adolescents. Obesity.

[84] Sawyer M.G., Harchak T., Wake M., Lynch J. (2011). Four-year prospective study of bmi and mental health problems in young children. Pediatrics.

[85] Wallander J.L., Taylor W.C., Grunbaum J.A., Franklin F.A., Harrison G.G., Kelder S.H., Schuster M.A. (2009). Weight status, quality of life, and self-concept in african american, hispanic, and white fifth-grade children. Obesity.

[86] Zeller M.H., Modi A.C. (2009). Development and initial validation of an obesity-specific quality-of-life measure for children: Sizing me up. Obesity.

